# Science and Old Medicine Contrasted

**Published:** 1891-12

**Authors:** T. F. Pomeroy

**Affiliations:** Providence, R. I.


### SCIENCE AND OLD MEDICINE CONTRASTED.

T. F. Pomeroy, A. M., M. D., Providence, R. I.

As ideas, like words, figures, chemical elements, and musical notes, are elementary and few in numbers as compared with the combinations of which they are susceptible, the difficulty of presenting those that are new is met at the threshold of an attempt to write an "original paper." This is peculiarly the case with a subject whose themes have long ago been exhausted, as is the fact with the one I have chosen for my paper on this occasion.

I may, however, avail myself of the capabilities for new combinations of old ideas with those of more recent date, in relation to subjects that are akin to that of medicine, notwithstanding the barrenness of ideas that has characterized the medical profession in relation to therapeutics, fully up to the commencement of the present century. For, while in all those branches of scientific investigation that are elementary and collateral to medicine, vast progress has been made, medicine itself, as an art, has been content to rest where the dark ages of the past had left it, so that to-day, even as then, the majority of its representatives are satisfied with the usages and with the methods, as they are with the means of cure that were then customary, and these are still the prevailing and popular ones upon which the great bulk of the human race relies in its utmost needs and under its sorest trials. This is due to those causes that have already been alluded to in a former paragraph, barrenness of ideas, as also to a neglect of those means of development, and of those processes of evolution that have insured the greater progress that has been made, both in science and art, everywhere but in medicine; those resources have evidently not been called into requisition in the cultivation of the medical art.

In mechanics, if we look at the steam engine of the past and of the present, we shall behold the great strides that have there been taken in the line of progress and improvement.

Some of us here can recall those primitive structures that were regarded with wonder and astonishment, as they were by steam propelled all along the course of the Hudson, and under the observation of the sparse populations contiguous to the great lakes not a very many years ago; and the first specimens of steam locomotion upon land, which the writer can well remember, within the past fifty-five years, that transported—in more senses than one—the passengers of those stage-coaching days from Albany to Schenectady, only sixteen miles of the journey to the then far West of Ohio and Michigan.

Look now at the magnificent and commodious steamships that traverse the wide ocean in every conceivable direction; regard the superb structure, that almost thing of life, the locomotive of the present day, with its long train of handsomely equipped and artistically constructed cars, supplied with every convenience and comfort that the weary or the exacting traveler could demand, and behold the march of progress. A progress that is the result of the evolution and the development of ideas. The first steamboat, the first locomotive were but, so to speak, the efflorescence, the flowering out of a simple idea, I might almost say, of the germ of an idea.

This physical manifestation of this first idea suggested new combinations of it with others that had already been made manifest and utilized, and so on from one improvement to another, until the present splendid triumphs of science and art, as applied to mechanics, have been thus reached.

An idea, a series of continually evolving ideas, were the germs, the seed about which all these results have clustered. Like the seeds of vegetation and their germs which supply themselves with nourishment from the elements that surround them, combining and arranging their particles in obedience to fixed laws, until we witness the magnificent forest, the prolific grains and fruits for the food of man and beast, and the beautiful flowers of the field and garden.

Who can tell how far apart are the ideas out of and from which such superb results of mechanical art have sprung, and the controlling principles and laws that determine the development and manifestations of organic life. In the construction of machinery are not living principles and eternal laws as truly operative and potent as in the more subtle and hidden processes that result in living organisms? May it not be that both series of results are due to a similar, if not to an identical relationship of cause and effect?

Both are indeed supplied and perfected from common elements as they are constructed and developed through the agency of common laws and universal principles.

Again, let us regard the vast attainments in science and art that have been made while medicine has thus remained stationary and dormant through its many years of hybernation, its sleep of centuries, and we will but glance at them, hardly more than to enumerate some few of them.

Compare the chemistry of the last with that of the present century, especially as applied to the arts and to kindred sciences; who would recognize the relationship from its present standpoint with that of the past? even within the memory of the writer it has almost passed recognition and comprehension. Then, the discoveries in astronomy and microscopy, and the revelations that are constantly made manifest through their agency; so also the vast and important advances in spectroscopic analyses and their results; the media of communication and inter-communication between points near and most remote furnished by telegraphy and its kindred agents; the processes of transferring, almost by magic, the images of objects, the symbols of thought, into tangible and convenient shape for use and ornament; the wonderful developments in the art of printing, and the great perfection attained in the construction of the printing press and its wonderful results and transformations. So also in biology —the science of life, the science of the sciences—have thought and research begun to bestir themselves, and ideas hitherto widely separated have commenced the processes of affiliation and of association, combining into definite forms, and into propositions, many of which await farther investigation and ultimate solution. Investigations and problems that reach back into the ages, that dig down into the hidden depths of the earth, that stretch forth into the spheres, that question as to the origin and history of the universe, that consult the very arcana of nature, and that stop at nothing that is between heaven and earth. Investigations that regard the most subtile, as well as the most material of the processes and manifestations of life, that relate to mental as to physical phenomena, that find analogies everywhere, and correspondences on every hand; in fact, that tend to unity and harmony, to universal similarity and relationship, to a grand incomprehensible central idea, the germ, the source of all things in the heavens above and in the earth beneath; that regard all organic life as but a microcosm, a representative of the universe itself, the outcome of an infinite series of evolutions and developments, obedient to the same eternal laws, subservient to the same subtile forces and constructed from the same elementary material. Such in general terms is the nature and direction of biological research, a science so vast, so comprehensible that it embraces all the rest within itself, one that cannot be regarded nor investigated without involving a knowledge that is universal, an apprehension that is eternal, a full comprehension of which must ever be unattainable.

It is "passing strange" that the medical art, the one whose relations are so exclusively confined to organic life, for the preservation and maintenance of its forces in equilibrium, and in the exercise of their highest capabilities should have been eminently the laggard in all that pertains to progress and development. It is astounding that it should have been an art so barren of ideas, one so destitute of a capacity for appropriating those of other arts and of the collateral sciences also, to its own use, and of recombining them for its own advancement. That such, however, is the fact cannot be controverted; almost daily and hourly does the evidence of it come under the notice of ordinary observation. But originality in the conception of ideas has not, nor ever has characterized the medical profession. It has rather been distinguished for its decided and persistent opposition to all such innovations, as are the outgrowth of original thought, as it has ever treated the authors of them with its disapproval, and not unfrequently, with persecution and a vindictiveness worthy of the bigotry that belongs only to ignorance and superstition.

While we may not be able fully to explain the causes that have led to these results, or to deny the facts or the history that records them, we are left to lament the consequences that an equally faithful history has also recorded, a history written not merely in books, but that is as indelibly stamped upon the victims of this ignorance and intolerance through a long succession of generations as upon the profession itself.

Why the medical profession did not, long before the present century, detect the intimate relations that exist between the three great kingdoms in nature, the mineral, the vegetable, and the animal, in relation to itself, why it has remained oblivious to the suggestions of nutrition and development incident to these relations, is a question, the solution of which has puzzled wiser heads than ours.

Why it has not from these facts of nutrition and growth, facts that have necessarily existed since the advent of organic life upon the earth, deduced a system of therapeutics commensurate with those relations is another cause for wonderment to those who, at this advanced period of the history of the world, have begun to enter upon the investigation and the practical application of them. From our standpoint the inference is most direct and legitimate that upon those laws that determine the facts and the phenomena of organic life must its continued existence and its healthful conditions depend; and that the same elementary constituents that enter into its construction, that administer to its nutrition and development, that maintain its functional action and direct its forces are requisite for the maintenance of their integrity and for the restoration of their harmonious action whenever disturbed or impaired, through disease or by accidental circumstances. Yet it has remained for representatives of the profession in this nineteenth century to make these deductions, and to announce this discovery, and to put them to the test of experience. And not only this, but in doing so to to meet the determined opposition, the unjust opprobrium and reproach of the great bulk of the profession, a reward for progressive research and advancement that has not been as liberally accorded to discovers in those sciences and arts that are collateral to and concurrent with the medical art. Well might the denunciations of one of old against the bigots and hypocrites of His day be hurled by them at their brethren and most unworthy representatives of the medical profession. "But woe unto you, scribes and pharisees, hypocrites! for ye shut up the kingdom of heaven against men; for ye neither go in yourselves, neither suffer ye them that are entering to go in." That these men who have had such a relish for pathological research, and who have wasted so much time and consumed so many volumes in their almost barren theories and speculations, should have failed to see the necessary connection, through the materia medica of nature, between physiological conditions and the requirements of therapeutics, while they were so keen on the pathological scent, is one of those bewildering things that meet us along the dreary pathway of medical science. Unhappily, too, the counterpart of this is found, far too largely found, in the midst of those who should know better, having themselves advanced, or assumed to do so, into a purer atmosphere of medical thought. Here also we are confronted by this absorbing and blinding bewilderment as to the paramount advantages of pathological research, the supreme importance of a *per se* knowledge of diseased states and conditions, apart from a perfect familiarity with the intimate relations existing between physiological and therapeutic ones.

Had an observation of every-day facts, in relation to health and disease, as constantly and as systematically commanded the attention of our professional ancestors as did their studies and lucubrations upon pathology in the abstract, the revelations of this our day as to therapeutic science would not have awaited the advent of the present century for their recognition and observance, nor would their reception have been as ungenerous and as ungracious as the history of that reception abundantly records.

Had the materia medica, which nature has always so profusely supplied and scattered along the pathway of the past ages, been studied in its relations to the physiological status; and had the results of its application thereto been as strictly observed and as faithfully recorded as during the latter years of the history of the medical art, we would not now have been compelled to the acknowledgment, the humiliating confession that medical science is far behind its contemporaries and its competitors in the race for scientific supremacy and advancement. Such obliviousness to their everywhere surroundings would require the almost logical inference that through all these ages of the past, and especially through these later years of progress, its members and the representatives of the medical profession, as a class, have not been the recipients of as thorough training, or of as full and complete education as their fellows and contemporaries in kindred scientific pursuits. How else shall we explain the fact that the suggestions derivable from mechanical and kindred forces, the study of which has always been prosecuted and enforced in all institutions of learning, have not been observed nor regarded in their application to medical science?

The subtle, the almost inscrutable power of the screw, the lever, and the pulley, the hidden, but most potent forces developed in the process of crystallization, of vegetable growth, and of the conversion of water into steam, to say nothing of those elementary forces, attraction and repulsion, would, or should be suggestive of the intimacy of their relations to animal life, and to the integrity of its healthful and continued existence. To the completely educated medical mind, and to the truly observant one, the human organism represents the sum of all the forces in nature, both those that are purely subtle and those that are merely mechanical; so also in the performance of its functions, voluntary or otherwise, he recognizes an implicit obedience to the same laws, and the same influences that govern the movements of the planets as also of the universe itself, for of these it is the legitimate and direct product, and upon these it is dependent for sustenance and growth, as well as for all the phenomena that characterize, or that relate to its existence. Mental function, which distinguishes the animal from that of all other manifestations of organic life, and most conspicuously in the human race, may, after all, be found to be but the highest form of force, the ultimate of the refining processes through which the forces of nature have progressed, the finality of many series of evolutions, the completion of the great circle of revolution that brings organized beings to their perihelion, to their nearest possible approach to that grand central force that governs and pervades all else in nature. At this point, in the order of nature, for the first time do we find, in kind though not in degree, a manifestation of attributes that belong only, so far as we are capable of understanding them, to the Deity itself, the great source of intelligence and of all things else. Here we must be content to rest, to be satisfied that we are animated by those forces, that we are the possessors of those faculties that make us capable of observing, and of investigating all phenomena that emanate or flow out from the great source of all things, from the divine mind itself.

We may congratulate ourselves, and feel happy over the thought that the medical profession, even with the rest of mankind, may yet aspire to the exercise of these functions, and indulge in their development whenever it shall awaken from its long period of inaction, its almost sleep of death, through which we may charitably suppose that, like the victims of its ignorance, it has been held under the influence and dominion of some demon of narcotism, of some infernal spell that bound it, body and soul, to the traditions and superstitions of the past in relation to medicine. If such reflections as these are pertinent, if such conclusions are just as regards the medical profession of the past, with how much more force and justice do they apply to the profession of this, our day, when knowledge stalks abroad and when science and art enjoy their holidays, and revel in the sunshine of their ever-fresh discoveries? Must the members of the profession, individually or collectively, rest content with the acquirements of past generations, yea, of past ages, with the methods of antiquity only at their command in their conflicts with disease, and especially at this juncture, when the horoscope of the astrologer, as the prognostications of the astronomer, alike point to the dire calamities of war, pestilence, and famine that have already begun to swell their onward tide, a tide which, before it ebbs again, may swallow up and destroy a tithe of the human race, and bring woe and desolation to millions more?

The great prodigality of nature in the production of life, which seems to spring spontaneously from everything and from everywhere, and which is so suggestive of her recuperative powers, is but the counterpart of her wastefulness and extravagance in the destruction of it.

Thus is put at naught, the great importance that is, by the human race centered in itself as the supreme end and object of all things else in nature, and for whose especial use and benefit the earth and all that it holds, the firmament and its myriads of shining orbs were definitely created and set in motion.

Such events, such great casualties as are just now foreshadowed serve to teach man that his race, in common with all others of the animal creation, is but an humble manifestation of nature's resources and capabilities, hidden away in this corner of the universe, and upon which the foot of old Time, as he passes this way, may but momentarily press to crush millions of its representatives out of sight and out of mind. We are also now and then reminded, and to ourselves most forcibly and painfully, that man's existence is no impediment to the onward march of the hurricane, or the resistless flow of the flood, no more than it for a moment retards the volcano's or the earthquake's relentless course; truly "all flesh is but grass," and we the creatures of a moment, and human existence but a flower that blooms to-day and to-morrow is dissolved into its elements, whose great prototype and exemplar is, nevertheless, the universe itself in its ever-changing and ever-varying course. Is it possible then that immortality, the immortality of which prophets and philosophers have written and speculated so much, of which the poets of all ages have sung, individual immortality, is but a dream of the imagination, a fantasy of the brain? Can it be that immortality appertains only to the perpetual evolution of the elements, and of the force of nature, alike through organic and inorganic matter, using them only for their manifestation and for the exhibition of their powers. The analogies and suggestions of all natural phenomena would almost lead to such a conclusion, as would also the lavishness of nature, both in the production and in the destruction of life, her utter unconcern and indifference as to the kind or the condition of it, whether vegetable or animal, or of a lower or a higher degree, it matters not; it is all the same.

Such problems as these do not yet admit of final conclusions, they must await the further developments of scientific investigation and the results of biological research, but, in regarding them solely from a scientific point of view, such may be the only alternative conclusions presented for our acceptance.

To no class of investigators, to no branch of scientists, do these investigations so properly belong as to those of the medical profession. The science of life in all its relations, and under all its conditions and manifestations, even to its final outcome, is the physician's appropriate field of action, the study of it his peculiar province.

